# Integrative analysis of copy number and transcriptional expression profiles in esophageal cancer to identify a novel driver gene for therapy

**DOI:** 10.1038/srep42060

**Published:** 2017-02-07

**Authors:** Gaochao Dong, Qixing Mao, Decai Yu, Yi Zhang, Mantang Qiu, Gaoyue Dong, Qiang Chen, Wenjie Xia, Jie Wang, Lin Xu, Feng Jiang

**Affiliations:** 1 Department of Thoracic Surgery, Jiangsu Key Laboratory of Molecular and Translational Cancer Research, Nanjing Medical University Affiliated Cancer Hospital, Cancer Institute of Jiangsu Province, Nanjing, Jiangsu, China; 2 Department of The Fourth Clinical College, Nanjing Medical University, Nanjing, Jiangsu, China; 3 Department of Hepatobiliary Surgery, The Affiliated Drum Tower Hospital of Nanjing University Medical College, Nanjing 210008, China; 4 Department of Thoracic Surgery, Xuzhou Centre Hospital, Xuzhou, Jiangsu, China

## Abstract

An increasing amount of evidence has highlighted the critical roles that copy number variants play in cancer progression. Here, we systematically analyzed the copy number alterations and differentially transcribed genes. Integrative analysis of the association between copy number variants and differential gene expression suggested that copy number variants will lead to aberrant expression of the corresponding genes. We performed a KEGG pathway and GO analysis, which revealed that cell cycle may have an effective role in the progression of esophageal cancer. FAM60A was then screened out as a potential prognostic factor through survival analysis and correlation analysis with clinical-pathological parameters. We subsequently showed that silencing of FAM60A could inhibit esophageal carcinoma tumor cell growth, migration and invasion *in vitro*. Through the bioinformatic analysis, we predict that FAM60A may act as a transcriptional factor to regulate genes that are correlated with each cell cycle. In summary, we comprehensively analyzed copy number segments and transcriptional expression profiles, which provided a novel approach to identify clinical biomarkers and therapeutic targets of esophageal carcinoma.

Esophageal cancer, classified as esophageal squamous cell carcinoma (ESCC) and esophageal adenocarcinoma (EAC), is the sixth leading cause of cancer death worldwide^1^,^2^. Currently, the standard therapy is still limited to surgical or endoscopic resection and chemoradiation^3^. In recent years, with the development of high-throughput next-generation sequencing (NGS) and genomic microarrays^4^, a considerable number of genes were found to be involved in initiation and development of esophageal cancer such as TP53, CDKN2A, FAT1, and PIK3CA^5^. Although advancements in diagnostics and therapeutics have improved clinical outcomes to some extent, the 5-year survival rate still remains 15–25%^6^. For earlier diagnosis and better treatment options in the future, we seek to identify new biomarkers and molecular targeted therapies.

Recent advances in genome characterization technologies have enabled systematic analysis to detect somatic copy number alterations (SCNA), which are distinguished from germline copy-number variations^7^,^8^. It has been reported that SCNA can affect oncogenic drivers which play a clear role in promoting cancer^9^,^10^. In some cases, SCNA have led to the identification of cancer-causing genes which emphasizes the important role of SCNA in cancer^11^,^12^. In oral cavity squamous cell carcinoma, amplifications of FGFR1 and PIK3CA were identified and were shown to be independent risk factors for prognosis^13^. In squamous cell lung carcinoma, copy number alteration of FGFR1, EGFR, HER2, PDGFRA, CCND1, SOX2 CDKN2A and PTEN were examined by fluorescence *in situ* hybridization^14^. It has been proposed that positive correlation of SCNAs with other genetic events may indicate functional synergies^15^,^16^. However, there has not been a large scale analysis concerned focusing on the relationship between SCNA and transcriptional gene expression profiles in esophageal cancer. This analysis will provide a novel approach to identify the oncogenes that were caused by alterations of copy number.

The Cancer Genome Atlas (TCGA) project makes it possible to analyze genomic and transcriptomic expression profiles simultaneously. With the help of the software R, we comprehensively analyzed the copy number alterations, as well as the different transcriptional gene expression profiles based on chip data from TCGA. The KEGG pathway enrichment analysis^17^ and GO biological process indicated that cell cycle had a substantial effect on the progress of esophageal cancer. Next, we integratively analyzed the copy number alterations and transcriptional gene variants and, selected abnormally expressed genes that were also involved in SCNA in a consistent direction. Among these genes, we found 53 associated with cell cycle, from which we determined that FAM60A expression is directly related to prognosis through the survival analysis (Fig. 1). The relationship between expression of FAM60A and clinical data were also explored. Additionally, the function of FAM60A was validated in two esophageal cancer cell line, Eca-109 and TE-13. Finally, the potential mechanism by which FAM60A regulates cell cycle was investigated. Collectively, up-regulation of FAM60A expression may result from amplification of the copy number alterations. Its high expression predicted a poor prognosis and was correlated with a malignant phenotype, which makes it a novel biomarker and a potential therapeutic drug target in the field of esophageal carcinoma.

## Results

### Comprehensive analysis of copy number alterations in esophageal carcinoma

**Figure 2:**
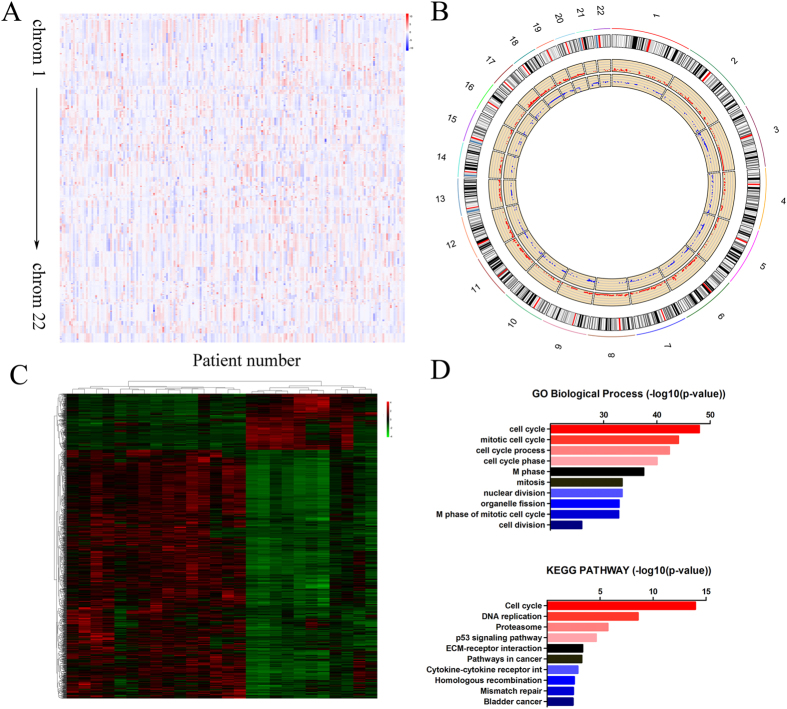
Systematic analysis of copy number alterations and differentially transcribed genes. (**A**) Copy number segment across esophageal carcinoma patients from TCGA. (**B**) CNTools and cghMCR packages of R software were used to identify regions related to copy number variant. The red region indicates amplification, while the blue region indicates deletion. (**C**) The heatmap reveals the significantly differentially expressed genes between tumor and normal samples. (**D**) Bioinformatic analysis of genes differentially expressed according to Gene Ontology and KEGG pathway.

To explore the genomic aberrations in esophageal cancer, we analyzed the chip data from TCGA database: TCGA_ESCA_GSNP6noCNV-2015-02-24 (delete germline CNV). There were 185 esophageal cancer patients included in the chip data (Fig. 2A). Using a CNTools package, we analyzed the copy number variants (Fig. 2A). In addition, we obtained the score of the gains or losses of copy number with a cghMCR package of R software (Fig. 2B). We identified 8607 genes for which the copy number was amplified and 3575 which were deleted. The top five amplifications or deletions of genes are listed in Table 1.

Our analysis of the copy number alterations showed that the most significant peak of amplification was located on the chromosome segment 6q23.3, which harbored MYB. MYB, which encodes a protein with three HTH DNA-binding domains, functions as a transcription regulator, and up-regulation has been shown to be an independent prognostic marker for esophageal carcinoma^18^. Additional amplification peaks with high significance were found on 6q23.2, 1p13.2, 17q12, and 12q11.21, which harbored SGK, NRAS, GRB7 and FAM60A. SGK and NRAS are recognized as oncogenes (Table 1). It has been reported that GRB7 is a driver gene associated with poor prognosis in esophageal cancer and which has an effect on the proliferation, migration and invasion capacities of cells^19^. However, the function of FAM60A in esophageal cancer has not been explored.

The most significant peaks of deletion were also identified on 20p11.21, 4p16.2, 5q12, 17p13.2 and 16p13.3. These regions harbor ZNF336, MSX1, NR2F1, CYB5D2 and NTHL1 respectively (Table 1). ZNF336, also known as GZF1, may regulate the spatial and temporal expression of the HOXA10 gene, which plays a role in morphogenesis^20^. MSX1 has been explored in many types of cancers and acts as a p53-interacting protein to regulate apoptosis of cancer cells^21^. In addition, NR2F1, an orphan nuclear receptor, has been confirmed to induce quiescence of disseminated tumor cells^22^. It has also been reported that CYB5D2 possesses tumor-suppressing activity and significantly inhibits cell invasion *in vitro* and cell-produced lung metastasis *in vivo*^23^. Additionally, the homozygous loss-of- function germline mutation in the NTHL1 gene is related to a new subtype of base-excision repair-associated adenomatous polyposis and colorectal cancer^24^.

### Comprehensive analysis of abnormally expressed genes in esophageal carcinoma

To identify potential esophageal cancer-related genes, we analyzed the chip data from TCGA database TCGA_ESCA_exp_HiSeq-2015-02-24. Information regarding 198 esophageal cancer patients was included in the data. In addition, there were 13 pairs of matched tumor and normal tissues. The limma package of R software was utilized to analyze the different transcriptional genes in the paired samples. Fold change >2 and and adjusted p-value of <0.05 were set to filter different genes, and we found 1374 genes that were aberrantly expressed significantly. Among these genes, 253 were down-regulated in tumors while 1121 were up-regulated (Fig. 2C). Next, we used Gene Ontology (GO) and KyotoEncyclopedia of Genes and Genomes (KEGG) pathway analysis (DAVID Bioinformatics Resources 6.7) to analyze the main function of the differentially expressed genes. As shown in Fig. 2D, the processes related to cell cycle achieved a high ranking in the enrichment analysis of GO Biological Processes. The same result was observed in the KEGG PATHWAY enrichment analysis, in which the top 4 were also associated with cell cycle (Fig. 2D). These results suggested that cell cycle may play an important role in the development of esophageal cancer, which is consistent with previous literature reports^5^,^25^.

### Integrative analysis of the association between copy number variants and differential gene expression

**Figure 3:**
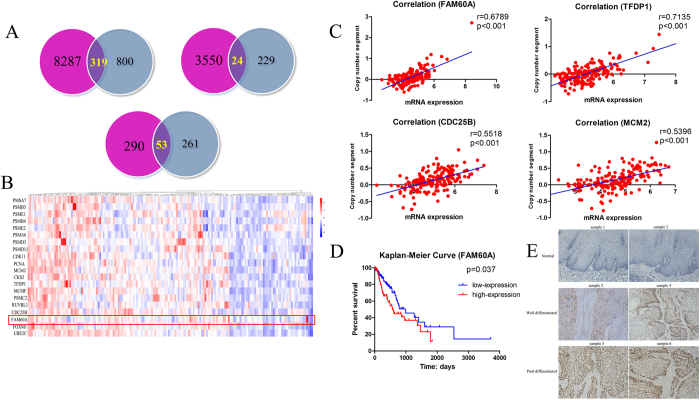
Integrative analysis of the association between copy number variant and differential gene expression. (**A**) Identification of differentially expressed genes related to cell cycle. (**B**) The top 20 expressed genes were related to cell cycle. (**C**) The correlation between copy number segment and the corresponding mRNA expression. (**D**) Survival analysis suggested FAM60A as a driver gene correlated with prognosis. (**E**) Representative images of immunohistochemical staining of FAM60A in normal esophageal tissues and different differentiated esophageal cancer tissues.

To explore the relationship between copy number variation and the transcriptional expression of significantly differentially expressed genes, we analyzed the genes both up-regulated in transcriptional expression and amplified in copy number as well as the genes down-regulated and deleted, and we found 319 genes and 24 genes, respectively (Fig. 3A). In the aforementioned enrichment analysis, we found that cell cycle may play an important role in the progression of esophageal cancer. Functional analysis identified 53 genes that were associated with cell cycle (Fig. 3A). Through the rank of the expression abundance, we selected the top 20 expressed genes (Fig. 3B). To verify the association between copy number variant and transcriptional expression of different genes, we calculated the correlation between copy number segment and the corresponding mRNA expression. As was expected, the segment of the copy number was highly correlated with the mRNA expression of the same gene, for example, genes such as FAM60A, TFDP1, CDC25B and MCM2, had correlation coefficients which were 0.6789, 0.7135, 0.5518 and 0.5396, respectively (Fig. 3C). It has been proved that those genes whose espressions correlated significantly with prognosis may function as oncogenes or suppressor genes in the development of cancer. Thus, we analyzed the impact of 20 genes with highest expression abundance on prognosis. We identified that FAM60A, the subunit of the SIN3-HDAC complex^26^,^27^, was a potential prognostic marker (Fig. 3D). Therefore, it was selected as the candidate driver gene for further research. We then selected two normal tissues and four tumor tissues to detect the protein expression of FAM60A using an immunohistochemical approach (Fig. 3E). Among the four tumor tissues, two of which were well differentiated, while the others were poorly differentiated. The expression of FAM60A was negative in the normal tissues while the expression of FAM60A was higher in the poorly differentiated tissues than in the well-differentiated tissues, indicating that FAM60A may function as an oncogene associated with malignant phenotype in cancers.

### Validation of the clinicopathological significance of FAM60A mRNA expression in ESCC

**Figure 4:**
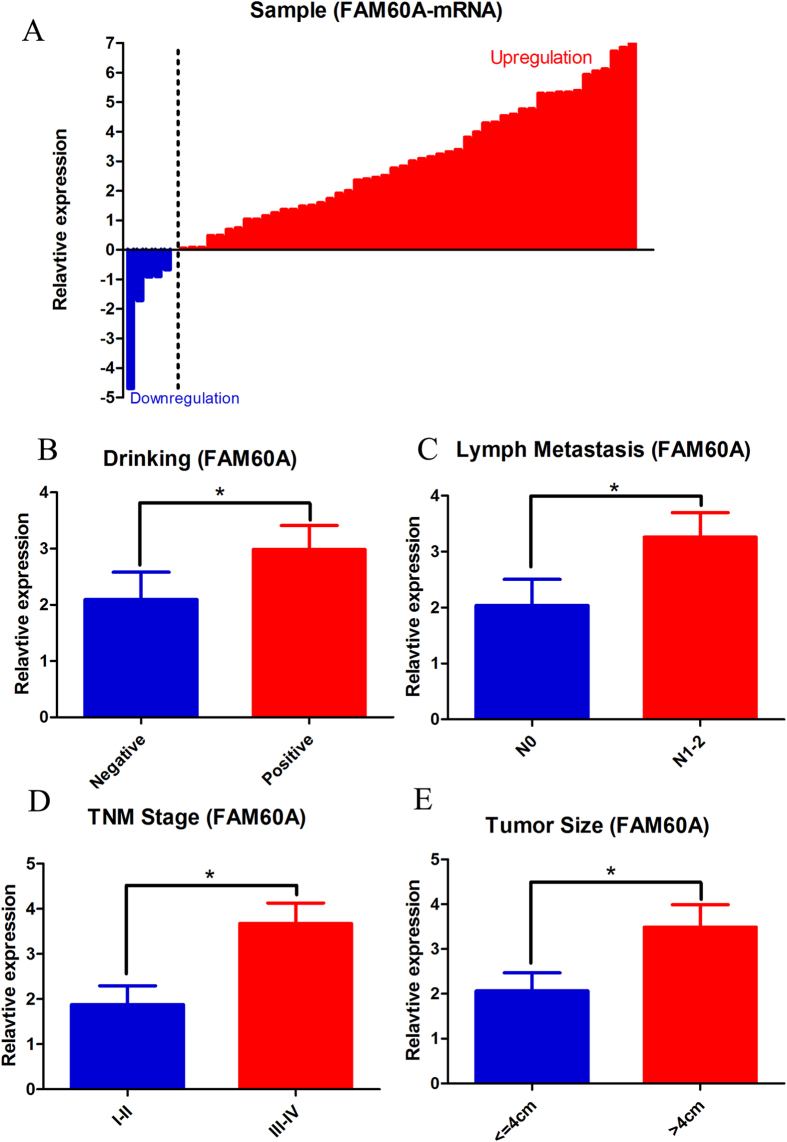
The expression of FAM60A in esophageal carcinoma patients. (**A**) qRT-PCR assay was performed to verify that FAM60A was up-regulated in tumor samples compared with matched normal tissues. (**B**–**E**) Indicate that the expression of FAM60A was positively related to tumor size, lymph node metastasis, TNM stage and alcohol consumption. The data are presented as the mean ± SD. *Indicates p < 0.05 versus the control group.

To further evaluate the clinical utility of FAM60A in the prognoses of esophageal cancer patients, its mRNA expression levels were detected by real-time RT-PCR in 55 paired tumor and non-tumor tissues with clinical data. Amazingly, we observed that mRNA expression of FAM60A was up-regulated in 50 tumor samples (90.9%) compared with normal samples (Fig. 4A). The median (2.53) of the relative expression was chosen to categorize the tumors into groups of high or low FAM60A mRNA levels. As shown in Table 2, correlation regression analysis indicated that high FAM60A mRNA levels were more common in patients who were drinkers (60.0 versus 30.0%, p = 0.032, Fig. 4B). In addition, the expression of FAM60A was positively correlated with tumor size (p = 0.043), lymph node metastasis (p = 0.022) and TNM stage (p = 0.022) (Fig. 4C–E). These results suggested that FAM60A over-expression may have an important role in disease progression of esophageal cancer.

### Validation of the function of FAM60A in esophageal cancer cell lines

**Figure 5:**
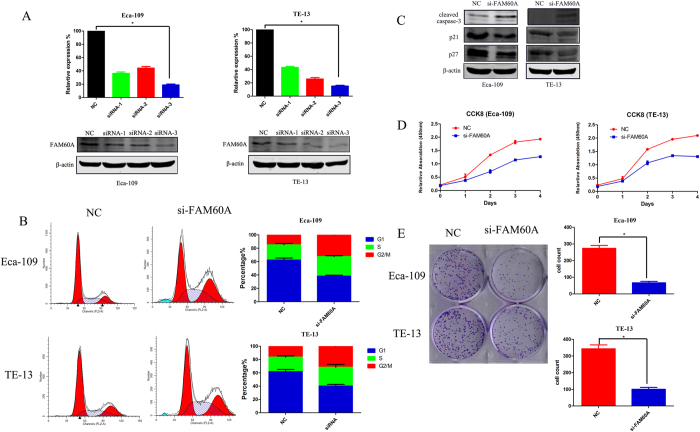
Knockdown of FAM60A expression in esophageal carcinoma cell lines. (**A**) Reductions in mRNA and protein levels of FAM60A 48 h after siRNA transfection in Eca-109 and TE-13 cells. (**B**) Cell cycle assay revealed that FAM60A inactivation decreased the percentage of G1 phase cells and arrested cells at G2/M phase. (**C**) Western blotting was utilized to detected the protein levels of cleaved caspase-3, p21 and p27 when the expression of FAM60A was suppressed. (**D**) CCK8 assay suggested that FAM60A promoted cell growth *in vitro*. (**E**) Clonogenic survival assay indicated that suppression of FAM60A inhibited the formation of clonogenic clusters. The data are presented as the mean ± SD. *Indicates statistical significance versus the control group (p < 0.05).

To explore the role of FAM60A in esophageal cancer cell lines, the expression of FAM60A was determined in Eca-109 and TE-13 cells, and found to be up-regulated (data not shown). Multiple RNA-interference sequences (siRNA-1 siRNA-2 and siRNA-3) were chosen to suppress the expression of FAM60A in cell lines. The reduction of FAM60A expression was confirmed by quantitative real-time RT-PCR (Fig. 5A). Transfection with the siRNA-3 sequence reduced the expression of FAM60A to nearly 20% in both of the two cell lines, which meant that siRNA-3 was the most efficient. The results were also confirmed by western blotting (Fig. 5A), which showed that the inhibition rate of siRNA-3 was the highest of the three siRNAs; therefore, we chose siRNA-3 for further experimental analyses.

**Figure 6:**
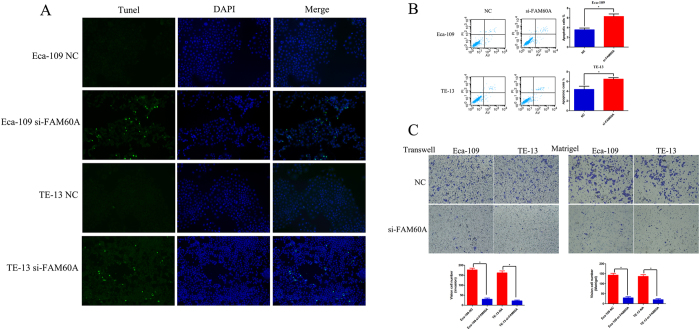
Function assay performed on esophageal carcinoma cell lines with knockdown of FAM60A. (**A**,**B**) TUNEL assay and flow cytometry analysis were performed and revealed that silencing of FAM60A increased apoptosis in esophageal carcinoma cell lines. (**C**) Matrigel and Transwell assays showed that the metastatic ability of Eca-109 and TE-13 cells was significantly inhibited by silencing of FAM60A. The data are presented as the mean ± SD. *Indicates p < 0.05 versus the control group.

First, we observed that knockdown of FAM60A expression had a significant effect on cell cycle (Fig. 5B), which was consistent with the previous results from analysis of KEGG pathway and GO terms. Suppression of FAM60A remarkably decreased the percentage of cells at G1 phase and increased the percentage of cells at G2/M phase compared with the control group. The protein expression levels of p21 and p27 were detected to confirm the cell cycle regulation, both of which are negative factors of G1 phase in the cell cycle (Fig. 5C). The results showed that inhibition of FAM60A expression suppressed the expression levels of p21 and p27, which is in accordance with the above results. Next, a cell-counting kit 8 (CCK-8) assay was chosen to explore the function of FAM60A in proliferation. siRNA-mediated knockdown of FAM60A reduced the proliferation of Eca-109 and TE-13 cells significantly compared with the negative control groups in both cell lines (Fig. 5D). A clonogenic survival assay was also performed, which indicated that suppression of FAM60A prominently reduced the numbers of clonogenic clusters (Fig. 5E). In addition, a TUNEL apoptosis assay was used to assess the influence of FAM60A on apoptosis. Knockdown of FAM60A showed that the number of fluorescent clusters increased significantly compared with the negative control groups in both cell lines, Eca-109 and TE-13 (Fig. 6A). Flowcytometry was also used to detect apoptosis in both cell lines. The results were consistent with the previous TUNEL apoptosis assay (Fig. 6B). The protein expression level of cleaved caspase-3 was also detected (Fig. 5C). The increased protein level of cleaved caspase-3 indicated that inhibition of FAM60A expression induced apoptosis. Finally, Transwell invasion assays and Matrigel assays were used to assess migration and invasion ability, respectively. The results indicated that the migration and invasion ability were both impaired in the FAM60A-knockdown cells (Fig. 6C). These results indicated that FAM60A plays an important role in cell cycle as well as proliferation and apoptosis. It may also influence migration and invasion, which is consistent with previous studies. FAM60A over-expression was significantly correlated with malignant phenotype in the cell lines, which is in agreement with the aforementioned findings regarding disease progression.

### The potential mechanism of FAM60A in esophageal cancer

**Figure 7:**
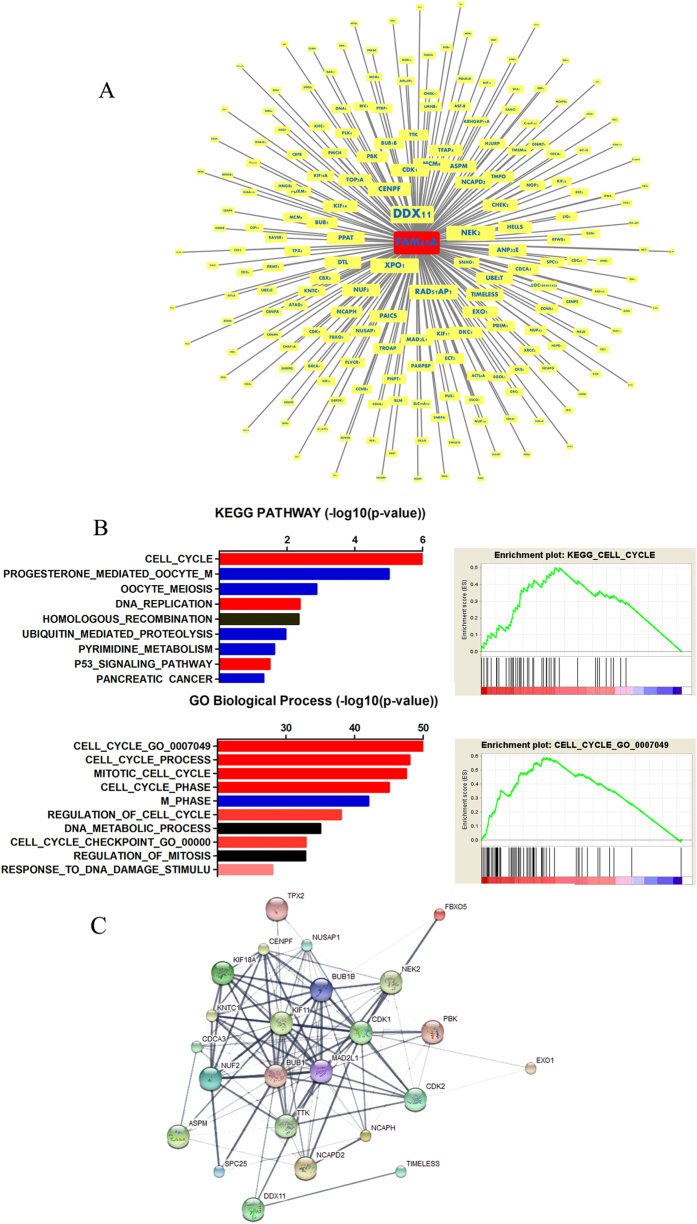
Bioinformatic analysis performed to predict the potential mechanism of FAM60A regulation of G2/M phase in the cell cycle. (**A**) The FAM60A co-expression genes. (**B**) GSEA enrichment analysis of the co-expressed genes. (**C**) SRTING was used to predict the protein network highly co-expressed with FAM60A.

To further explore the potential mechanism by which FAM60A regulates the malignant phenotype, we analyzed the correlation of FAM60A with significantly differentially transcribed genes (1372 genes) by the CORREL function (Fig. 7A, showed 200 genes). We observed that expression of DDX11 was mostly relevant to that of FAM60A, which is a protein-coding gene related to nucleic acid binding and double-stranded DNA binding. By deleting correlation coefficients less than 0.3 or greater than −0.2, we obtained 719 genes. GSEA was used to do KEGG pathway and GO terms enrichment analysis, which is a powerful tool to infer gene function. As shown in Fig. 7B, the enrichment of cell cycle ranked as number 1 both in KEGG pathway and GO terms. In previous studies^26^,^27^, it has been shown that FAM60A bounded SIN-HDAC complex through a conserved GATA-like zinc finger, which regulates gene expression. In the aforementioned experimental results, knock-down of FAM60A expression decreased the percentage of cells at G1 phase and arrested cancer cells at G2/M phase. Thus, we hypothesized that FAM60A may act as a transcription factor to regulate genes that are correlated with cell cycle. Through the GO terms analysis of the significant different transcriptional genes, we got the 100 genes which correlated with the G2/M phase. Through the STRING functional protein association networks, we obtained results that related the protein interactions (Fig. 7C). In this network, we found 24 proteins that have interactions with other proteins. FAM60A may regulate the expression of KIF11, BUB1, and MAD2L1, which may serve important roles in controlling the cell cycle. KIF11, is a motor protein that belongs to the kinesin-like protein family, the function of which includes establishing a bipolar spindle during cell mitosis. BUB1, a serine/threonine-protein kinase, plays a central role in mitosis. MAD2L1, a component of the spindle-assembly checkpoint, monitors the progress of kinetochore-spindle attachment and inhibits the activity of the anaphase-promoting complex. They may serve as candidate genes that FAM60A acts as the transcriptional factor to regulate.

## Discussion

In this study, we comprehensively analyzed the copy number alterations in esophageal carcinoma as well as the differential transcriptional gene expression between normal and tumor specimens. We identified that the regions of 6q23.3, 6q23.2, 1p13.2, 17q12, and 12q11.21 show amplifications; however, the regions of 20p11.21, 4p16.2, 5q12, 17p13.2, and 16p13.3 show deletions. There are some oncogenes and tumor suppressor genes that had been reported previously to be located in these regions, such as NRAS, GRB7, ZNF336, MSX1. In addition, we identified 1374 differentially expressed genes between tumor and matched normal specimens. Bioinformatic analysis indicated that cell cycle may play an important role in the development of esophageal carcinoma. Interestingly, we found that there is an intimate relationship between the copy number segment and the corresponding mRNA expression, which told us that copy number variants may have an influence on gene expression. Through the survival analysis, FAM60A was selected as a candidate gene that had a significant correlation with prognosis. Through a qRT-PCR assay, we verified that FAM60A was up-regulated in esophageal carcinoma patients, which was positively correlated with tumor size, lymph node metastasis, TNM stage and alcohol consumption. The function assay revealed that knock-down of FAM60A expression could decrease the percentage of G1 phase cells, arrest cells at G2/M phase, suppress cell proliferation and increase apoptosis. It also depressed the migration and invasion ability of cells *in vitro*. Finally, we predicted that FAM60A may serve as a transcriptional factor to regulate candidate genes such as KIF11, BUB1, and MAD2L1, which play important roles in cell cycle regulation. These data confirmed that FAM60A promotes esophageal tumor progression and is related to prognosis, suggesting that FAM60A could be a therapeutic target and biomarker.

It has been demonstrated that copy number variations affect larger fractions of the genome in cancer than do other type of somatic genetic alterations. Understanding the mechanisms affecting the corresponding gene expression in cancer might contribute to understanding biological differences and help to identify new therapeutic targets. Through comprehensive genomic analysis of 158 ESCC cases, FAM135B was identified as a novel cancer-implicated gene that promoted malignancy of ESCC cells^5^. MIR548K, a microRNA encoded in the amplified 11q13.3–13.4 region, was also characterized as a novel oncogene that enhanced malignant phenotypes of ESCC cells. A single-nucleotide polymorphism array-based copy number profile was performed and revealed that focal amplifications of YAPA and loss-of-function mutations in FAT1 and AJUBA may contribute to the activation of WNT signaling in ESCC^12^. Whole-genome sequencing and whole-exome sequencing of patients with different stages of ESCC were performed and revealed that somatic amplifications at 8q were enriched in stage I tumors, and deletions of 4p-q and 5q were specifically identified in stage III tumors^28^. FAM84B was also identified as a candidate oncogene that was amplified and influenced cell growth, migration and invasion in ESCC cell lines. Copy number alterations were also analyzed among esophageal adenocarcinoma, which showed that SKI and PRKCZ, biomarkers involved in transforming growth factor-β pathway, were located at a deletion region, suggesting the potential utility of novel biomarkers for EA^29^. Genome-wide copy number variation analysis was performed on ESCC samples and identified that amplification of ABCC4 located at 13q32.1 was significantly correlated with ESCC risk, which was an independent poor prognostic factor for ESCC^30^.

There are few studies focused on the biological function of FAM60A^26^,^27^,^31^. Ivan M. Muñoz and colleagues found that FAM60A, a cell cycle-regulated protein, could bind to the SIN3-HDAC complex which deacetylates histones, thereby repressing gene transcription. Quantitative proteomics also determined that FAM60A is the subunit of the Sin3 deacetylase, which regulates the expression of genes that encode components of the TGF-beta signaling pathway. However, the functions of FAM60A revealed in lung cancer and liver cancer cells suggest that FAM60A may act as a tumor-suppressing gene. This is not consistent with this study, which indicats that FAM60A may have different roles across diverse cancers.

Overall, this study provides important insights into copy number variants of esophageal carcinoma, and FAM60A was demonstrated as a driver gene in esophageal carcinoma that acts as a new therapeutic target. This comprehensive analysis of genomic and transcriptional data emphasized that copy number variations play important roles in the progression of cancer which provides a novel approach for the treatment of esophageal carcinoma.

## Materials and Methods

### Data sources and bioinformatics

The level 3 TCGA data TCGA_ESCA_GSNP6noCNV-2015-02-24 (delete germline CNV) and TCGA_ESCA_exp_HiSeq-2015-02-24 were downloaded from the website of the UCSC cancer browser (http://genome-cancer.ucsc.edu) and contain 185 esophageal cancer samples and 198 esophageal cancer samples, respectively. The packages of CNTools and cghMCR in software R were used to identify the significant peak of copy number variants. The limma software package was also used to reveal the differential transcriptional gene expression between normal and tumor specimens. The list of different transcriptional genes was submitted to DAVID Bioinformatics Resources 6.7 (http://david.abcc.ncifcrf.gov) for KEGG pathway and GO biological process enrichment analysis. The GSEA was performed by GSEA software and gene sets used in this work were downloaded from the Molecular Signatures Database (http://software.broadinstitute.org/gsea/msigdb/index.jsp, MSigDB v4.0, released Jun 7, 2013).

### Patient and tissue samples

This research was approved by the Ethics Committee of the Cancer Institute of Jiangsu Province. Between 2012 and 2013, 55 paired tumor tissues and matched non-tumor tissues were obtained from esophageal cancer patients who received surgical resection at the department of thoracic surgery, Cancer Institute of Jiangsu Province, China. All surgical specimens were stored in liquid nitrogen immediately after resection until total RNA extraction. Two pathologists performed the histopathological classifications in a double-blind fashion. At least 80% of the tumor samples were composed of viable-appearing tumor cells on histological assessment. All patients in this research had signed informed consents. The methods performed in this study were in accordance with the guidelines and regulations of the Ethics Committee of the Cancer Institute of Jiangsu Province.

### Immunohistochemistry

Tissue sections were deparaffinized and rehydrated through graded alcohol. Endogenous peroxidase activity was blocked by incubation in 3% H_2_O_2_. Antigen retrieval was performed with 0.01 M citrate buffer (pH 6.0) and microwave heat induction. An anti-FAM60A rabbit polyclonal antibody (Abcam, ab122444 1:50) was used.

### Cell culture and siRNA transfection

The human esophageal squamous cell carcinoma cell lines Eca-109 and TE-13 were purchased from the Chinese Academy of Science (Shanghai, China). The cells were maintained in DMEM (Dulbecco’s Modified Eagle’s Medium, GIBCO) with 10% FBS (fetal bovine serum, GIBCO), 100 U/ml penicillin and 100 mg/ml streptomycin (KeyGEN, Nanjing, China) at 37 °C under a humidified atmosphere of 5% CO_2_. Eca-109 and TE-13 cells were transfected with small interfering RNAs (siRNAs) or negative control sequences using iMAX (Invitrogen, Shanghai, China). Three sequences were designed and the sequences were as follows: siRNA-1 for FAM60A: sense 5′-GCAACCAGAUCAGUAAACUTT-3′, antisense 5′-AGUUUACUGAUCUGGUUGCTT-3′; siRNA-2 for FAM60A: sense 5′-CUGGAAUCAUGUGGUAGAUTT-3′, antisense 5′-AUCUACCACAUGAUUCCAGTT-3′; siRNA-3 for FAM60A: sense 5′-CUGACAGUAAACGCUAUGATT-3′, antisense 5′-UCAUAGCGUUUACUGUCAGTT-3′.

### Total RNA extraction and qRT-PCR analysis

The total RNA was extracted from tissues or cultured cells with TRIzol reagent (Invitrogen, Carlsbad, CA, USA), according to the manufacturer’s protocol. For RT-PCR, 2000 ng of total RNA was reverse-transcribed to a final volume of 20 μl cDNA using a Reverse Transcription Kit (Takara, cat: RR036A). qRT-PCRanalyses were performed with SYBR Select Master Mix (AppliedBiosystems, cat: 4472908) using the QuantStudioTM 6 Flex Real-Time PCR system. The qRT-PCR reaction steps included an initial denaturation step at 95 °C for 10 min, followed by 40 cycles of 92 °C for 15 sec and 60 °C for 1 min. The qRT-PCR primers for FAM60A and β-actin were as follows: primers for FAM60A, forward primer 5′-CTCCAGTTCTCGATTCACTGAC-3′, and reverse primer 5′-CGAGTCTCATGCAATCCAAAACA-3′; primers for β-actin: forward primer 5′-CCCTGGTCAAATTGCTTAACCT-3′ and reverse primer: 5′-TTATTCGTCCCTCTGTTTTATGGAT-3′. The Ct value for each sample was calculated with the ΔΔCt-method, and the fold expression changes (tumor versus normal) were calculated using 2^−ΔΔCT^ methods.

### Western blotting assay

Cells were seeded in 6-well plates and transfected with siRNAs or negative control sequences as previously described. The cells were washed with ice-cold PBS, lysed with RIPA buffer (KeyGEN) and centrifuged (14000 rpm, 10 min, 4 °C). The protein concentration was measured using a BCA Assay Kit (KeyGEN). Western blot analysis was performed as previously described^32^ used the following antibodies: FAM60A (Abcam, ab167180, 1:500), p27 (Abcam, ab545569, 1:500), p21 (CST, 2947, 1:1000), caspase-3 (CST, 9665, 1:1000), β-actin (CST, 12262, 1:1000).

### TUNEL assay

Eca-109 and TE-13 cells were seeded on coverslips. After 48 h of transfection with si-FAM60A or NC sequences, cells were fixed in 4% paraformaldehyde for 15 min at room temperature. Cell apoptosis was detected using a Terminal Deoxynucleotidyl Transferase-Mediated dUTP Nick-End Labelling (TUNEL) assay kit (Kaygene, Nanjing, China) according to the manufacturer’s instructions. Cells were then washed and stained with DAPI. Coverslips were mounted onto glass slides using a Zeiss Axioscope inverted fluorescence microscope (Zeiss, Axio Vert. A1).

### Flow cytometry analysis

Eca109 and TE-13 cells were seeded in a 6-well plate at an appropriate density and were harvested after 48 h of transfection with si-FAM60A or NC sequences, respectively. The cells were washed with ice-cold PBS and fixed with ice-cold 70% ethanol. After storage at −20 °C, the cells were washed with PBS and redistributed in 0.5 ml of propidium iodide (PI)/RNase staining buffer (BD bioscience, USA) for 30 min at room temperature in the dark. Cell cycle distribution assessment was performed with a FACS Calibur flow cytometer (BD bioscience, USA). The results shown arerepresentative of at least three separate experiments.

The Annexin V-FITC and PI binding assay (KeyGEN Biotech, Nanjing, China) was performed to assess apoptosis and necrosis of cells *in vitro*. Eca109 and TE-13 cells were seeded in a 6-well plate at an appropriate density. After 48 h of transfection with si-FAM60A or NC sequences, the cells were trypsinized, washed in PBS and resuspended in 100 μl of binding buffer after a 24 h incubation. Before analysis, the cells were cultured in the dark for 15 min with 10 μl of Annexin V-FITC and 10 μ of PI, and then, 400 μl binding buffer was added to the culture. Apoptosis and necrosis were assessed with a FACS Caliburflowcytometer (BD bioscience, USA). Each experiment was performed in triplicate.

### CCK8 assay

Eca109 and TE-13 cells were seeded in a 6-well plate at an appropriate density and were harvested after 48 h of transfection with si-FAM60A or NC sequences, respectively. Cells from both cell lines were seeded into 96-well plates (3000/well) and incubated in DMEM at 37 °C and 5% CO_2_ atmosphere. The Cell Counting Kit-8 assay was used to detect relative cell growth according to the manufacturer’s instructions. The absorbance was measured at 450 nm with an ELx-800 Universal Microplate Reader. Each experiment was repeated at least three times independently.

### Clonogenic assay

A total of 400 transfected cells were placed in a fresh six-well plate and maintained in medium containing 10% FBS for colony formation assays. After one week, the medium was replaced. Two weeks later, cells were fixed with 4% paraformaldehyde and stained with 0.1% crystal violet. Visible colonies were manually counted; and each experiment was repeated three times.

### Transwell and Matrigel assays

For the Transwell assay, transfected cells (40000 cells in 100 μl per well) were seeded in the upper chamber of Transwell assay inserts (8 mm pores, Millipore, Billerica, MA), which contained 200 μl of serum-free DMEM. The 500 μl of DMEM containing 10% FBS was loaded into the lower chambers. After 24 h of incubation at 37 °C in a 5% CO_2_ atmosphere, the cells on the upper chamber filter surface were fixed with 4% paraformaldehyde and stained with 0.1% crystal violet. Migration was evaluated by counting the number of stained cell in five random fields per filter in each group.

For the Matrigel assay, transfected cells (50000 cells in 100 μl per well) were plated in the upper chamber with a Matrigel-coated membrane (BD Biosciences) in 300 μl of serum-free DMEM. The bottom chambers contained DMEM with 10% FBS. The cells were harvested after a 48 h incubation at 37 °C in a 5% CO_2_ atmosphere.

### Statistical analysis

All experiments were representative of at least three trials and the data were expressed as the mean** ± **SD. Significant differences between the groups were analyzed using Student’s *t*-test. Statistical analysis was performed using SPSS (version 17.0; SPSS, Inc.) and *p* < 0.05 was considered statistically significant.

## Additional Information

**How to cite this article**: Dong, G. *et al*. Integrative analysis of copy number and transcriptional expression profiles in esophageal cancer to identify a novel driver gene for therapy. *Sci. Rep.* **7**, 42060; doi: 10.1038/srep42060 (2017).

**Publisher's note:** Springer Nature remains neutral with regard to jurisdictional claims in published maps and institutional affiliations.

## Figures and Tables

**Figure 1:**
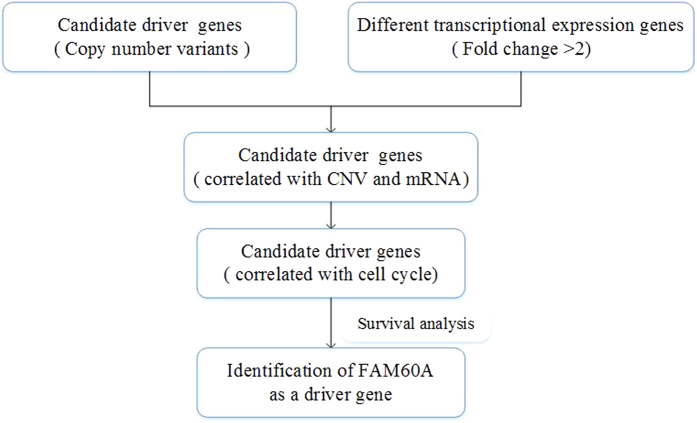
Flowchart for comprehensive analysis of the copy number and transcriptional expression profiles and identification of the FAM60A as a driver gene correlated with prognosis.

**Table 1:** Candidate driver genes correlated with copy number variants.

Gene	Locus	Amplification/Deletion	Gains/Losses Score
MYB	6q23.3	Amplification	4.0729
SGK	6q23.2	Amplification	3.9074
NRAS	1p13.2	Amplification	2.6600
GRB7	17q12	Amplification	2.5662
FAM60A	12q11.21	Amplification	1.9742
ZNF336	20p11.21	Deletion	−4.6088
MSX1	4p16.2	Deletion	−3.7249
NR2F1	5q12	Deletion	−3.2108
CYB5D2	17p13.2	Deletion	−3.0895
NTHL1	16p13.3	Deletion	−3.0598

**Table 2:** Correlation between FAM60A expression and clinicopathologial characteristics.

Variable	Cases (n)	Expression level of FAM60A		p-value	Pearson(χ^2^)
		Low (n, %)	High (n, %)		
Age					
<60	20	10 (50.0)	10 (50.0)	0.919	0.010
≥60	35	18 (51.4)	17 (48.6)		
Drinking					
Yes	35	14 (40.0)	21 (60.0)	0.032*	**4.583**
No	20	14 (70.0)	6 (30.0)		
Smoking					
Yes	13	8 (61.5)	5 (38.5)	0.380	0.770
No	42	20 (47.6)	22 (52.4)		
Differentiation					
Well	7	4 (57.1)	3 (42.9)	0.444	1.625
Moderate	32	14 (43.8)	18 (56.2)		
Poor	16	10 (62.5)	6 (37.5)		
Tumor location					
Upper	8	3 (37.5)	5 (62.5)	0.686	0.755
Middle	33	18 (54.5)	15 (45.5)		
Lower	14	7 (50.0)	7 (50.0)		
Tumor size					
≤4 cm	32	20 (62.5)	12 (37.5)	0.043*	**4.114**
>4 cm	23	8 (34.8)	15 (65.2)		
Lymph node					
Negative	27	18 (66.7)	9 (33.3)	0.022*	**5.269**
Positive	28	10 (35.7)	18 (64.3)		
TNM staging					
I–II	31	20 (64.5)	11 (35.5)	0.022*	**5.263**
III–IV	24	8 (33.3)	(66.7)		

*Indicates p < 0.05.
